# An Unusual Case of Giant Cell Arteritis (GCA) Presenting With Bilateral Visual Loss

**DOI:** 10.7759/cureus.106206

**Published:** 2026-03-31

**Authors:** Kevin C Dsouza, Mohamed H Riyal, Krupa Abraham, Jordan Chow, Avani Bhingradiya

**Affiliations:** 1 Medicine, Bedfordshire Hospitals NHS Foundation Trust, Bedford, GBR; 2 Acute Medicine, Bedfordshire Hospitals NHS Foundation Trust, Bedford, GBR; 3 General Internal Medicine, Bedfordshire Hospitals NHS Foundation Trust, Bedford, GBR; 4 General Medicine, Bedfordshire Hospitals NHS Foundation Trust, Bedford, GBR

**Keywords:** anterior ischemic optic neuropathy, atypical presentation, bilateral visual loss, central retinal artery occlusion, corticosteroid therapy, elderly patients, giant cell arteritis, halo sign, skip lesions, temporal artery ultrasound

## Abstract

We report the case of a 77-year-old woman who presented with progressive, painless bilateral visual loss over three weeks. Ophthalmologic evaluation demonstrated bilateral anterior ischemic optic neuropathy and right central retinal artery occlusion. Notably, she lacked classical systemic features of giant cell arteritis (GCA), including headache, scalp tenderness, or jaw claudication. Laboratory evaluation revealed an elevated erythrocyte sedimentation rate (ESR 64 mm/h). Temporal artery ultrasound demonstrated bilateral halo signs, supporting the diagnosis of GCA. High-dose corticosteroid therapy was promptly initiated; however, visual loss remained irreversible.

This case highlights the importance of maintaining a high index of suspicion for GCA in elderly patients presenting with unexplained visual symptoms, even in the absence of typical cranial features. Temporal artery ultrasound is a valuable non-invasive diagnostic tool that can facilitate early diagnosis and prompt treatment, potentially preventing irreversible visual complications.

## Introduction

Giant cell arteritis (GCA) is the most common systemic vasculitis affecting adults over the age of 50 years, with a predilection for women and individuals of Northern European descent [1,2]. The incidence is estimated at 15-25 cases per 100,000 persons aged over 50 years, and despite advances in diagnostic pathways, irreversible visual loss remains a significant complication in untreated patients [2,3]. Classical clinical features include temporal headache, jaw claudication, scalp tenderness, and elevated inflammatory markers [2]. However, GCA can present atypically, particularly in elderly patients who may lack classic cranial symptoms, leading to delayed diagnosis and an increased risk of visual complications, including bilateral visual loss [3]. Early recognition is therefore crucial, and vascular imaging, particularly temporal artery ultrasound, has emerged as an important non-invasive diagnostic modality for evaluating suspected GCA and is sometimes considered over temporal artery biopsy [1,4-7].

This case highlights a rare and atypical presentation of GCA with progressive bilateral visual loss occurring in the absence of classic systemic symptoms, underscoring the importance of maintaining a high index of suspicion in elderly patients with unexplained visual impairment.

## Case presentation

This case describes an atypical and sight-threatening presentation of GCA in an elderly patient presenting with rapidly progressive visual loss. 

A 77-year-old woman with a history of osteoarthritis, chronic plaque psoriasis, hypertension, type 2 diabetes mellitus, collagenous colitis, and a prior transient ischemic attack presented to the emergency department with a three-week history of gradual, painless visual loss initially affecting her left eye. She denied headache, scalp tenderness, jaw claudication, or constitutional symptoms.

On presentation in the emergency department, ophthalmologic assessment demonstrated normal orbital appearance and full extraocular movements. Visual acuity was 6/6 in the right eye and no perception of light (visual acuity 0) in the left eye. There was no evidence of visual neglect on examination. Pupillary responses were not formally documented at presentation. The patient was discussed with the on-call ophthalmologist and arranged for urgent outpatient ophthalmology review, where she was diagnosed with a left retinal branch occlusion and cataract. Carotid Doppler ultrasound performed shortly thereafter did not demonstrate any significant stenosis or vascular abnormality.

Eleven days later, she presented again to the emergency department with worsening visual loss in the right eye and complete loss of vision in the left eye. A head CT was performed, and did not demonstrate any acute intracranial pathology. She was again referred to ophthalmology, where further assessment demonstrated bilateral anterior ischemic optic neuropathy and a right central retinal artery occlusion.

In view of the rapid progression of bilateral visual loss, she was admitted under the medical team for further evaluation.

On presentation, she was reviewed by the medical team, and laboratory investigations demonstrated elevated inflammatory markers, including a markedly raised erythrocyte sedimentation rate and C-reactive protein, supporting an inflammatory process (Table 1). Several laboratory abnormalities, including hypoalbuminemia, elevated immunoglobulin levels, and raised serum free light chains, were noted. These findings were considered consistent with a non-specific inflammatory response rather than a primary pathological process contributing to the patient’s visual presentation. There was no evidence of an alternative hematological or autoimmune condition to explain these abnormalities.

**Table 1:** Laboratory investigations Reference ranges are based on standard adult UK laboratory values.

Test	May 13, 2025	May 21, 2025	Reference range
C-reactive protein (CRP)	35	3.3	<5 mg/L
Erythrocyte sedimentation rate (ESR)	64	N/A	<30 mm/h
White cell count (WCC)	7.6	11.9	4.0-11.0 × 10⁹/L
Neutrophils	5.49	7.85	2.0-7.5 × 10⁹/L
Hemoglobin	126	135	115-160 g/L
Platelets	332	226	150-400 × 10⁹/L
Sodium	136	141	135-145 mmol/L
Potassium	4.4	4.6	3.5-5.0 mmol/L
Urea	4.9	N/A	2.5-7.8 mmol/L
Creatinine	43	41	45-84 µmol/L
Alkaline phosphatase (ALP)	164	90	30-130 IU/L
Alanine aminotransferase (ALT)	17	17	5-40 IU/L
Total bilirubin	7	4	<21 µmol/L
Albumin	26	23	35-50 g/L
Serum kappa free light chain	66	N/A	3.3-19.4 mg/L
Serum lambda free light chain	35.3	N/A	5.7-26.3 mg/L
Kappa/lambda ratio	1.87	N/A	0.26-1.65
Rheumatoid factor (RF)	25.6	N/A	<20 IU/mL
Immunoglobulin G (IgG)	19.11	N/A	7.0-16.0 g/L
Immunoglobulin A (IgA)	3.5	N/A	0.7-4.0 g/L
Immunoglobulin M (IgM)	3.65	N/A	0.4-2.3 g/L
Paraprotein	None detected	N/A	Not detected

Differential diagnoses considered included embolic phenomena, intracranial pathology, and other causes of optic neuropathy. However, the absence of cardioembolic sources on echocardiography, normal neuroimaging, and lack of large-vessel pathology on CT angiography made these less likely. In contrast, the presence of bilateral anterior ischemic optic neuropathy in conjunction with elevated inflammatory markers supported an inflammatory etiology, with temporal artery ultrasound findings confirming the diagnosis of GCA. The patient’s Southend Giant Cell Arteritis Probability Score indicated a high probability of disease.

In view of the high clinical suspicion and risk of irreversible visual loss, high-dose corticosteroid therapy was initiated immediately prior to definitive diagnostic confirmation. The patient received intravenous methylprednisolone once a day for three days, followed by high-dose oral prednisolone.

Following initiation of treatment, a series of investigations were undertaken to exclude alternative causes of bilateral visual loss. Fundoscopy demonstrated bilateral anterior ischemic optic neuropathy. Cross-sectional and vascular imaging, including CT of the head, MRI of the brain, echocardiography, and CT aortography, did not demonstrate intracranial pathology, cardioembolic sources, or large-vessel vasculitis (Table 2).

**Table 2:** Imaging and diagnostic investigations This table summarizes key imaging and diagnostic investigations performed during the patient’s admission. Reference findings indicate expected normal or negative results used for comparison when interpreting investigations relevant to giant cell arteritis.

Investigation	Date	Findings	Reference/normal findings
Carotid Doppler ultrasound	01/05/2025	No significant carotid artery stenosis	No hemodynamically significant stenosis
Fundoscopy	13/05/2025	Bilateral anterior ischemic optic neuropathy	Normal optic disc appearance
CT head	13/05/2025	No acute intracranial abnormality	No acute hemorrhage or infarction
CT aortogram	13/05/2025	Normal thoracic and abdominal aorta; no aortitis	No aortic dilatation, dissection, or wall inflammation
Echocardiography	14/05/2025	Preserved left ventricular systolic function; no thrombus	Normal ventricular function; no intracardiac thrombus
MRI brain	15/05/2025	No acute infarction; chronic microvascular changes	No acute ischemia or structural abnormality
Temporal artery ultrasound	15/05/2025	Bilateral halo signs with circumferential wall thickening	No halo sign; normal intima-media thickness

**Figure 1:**
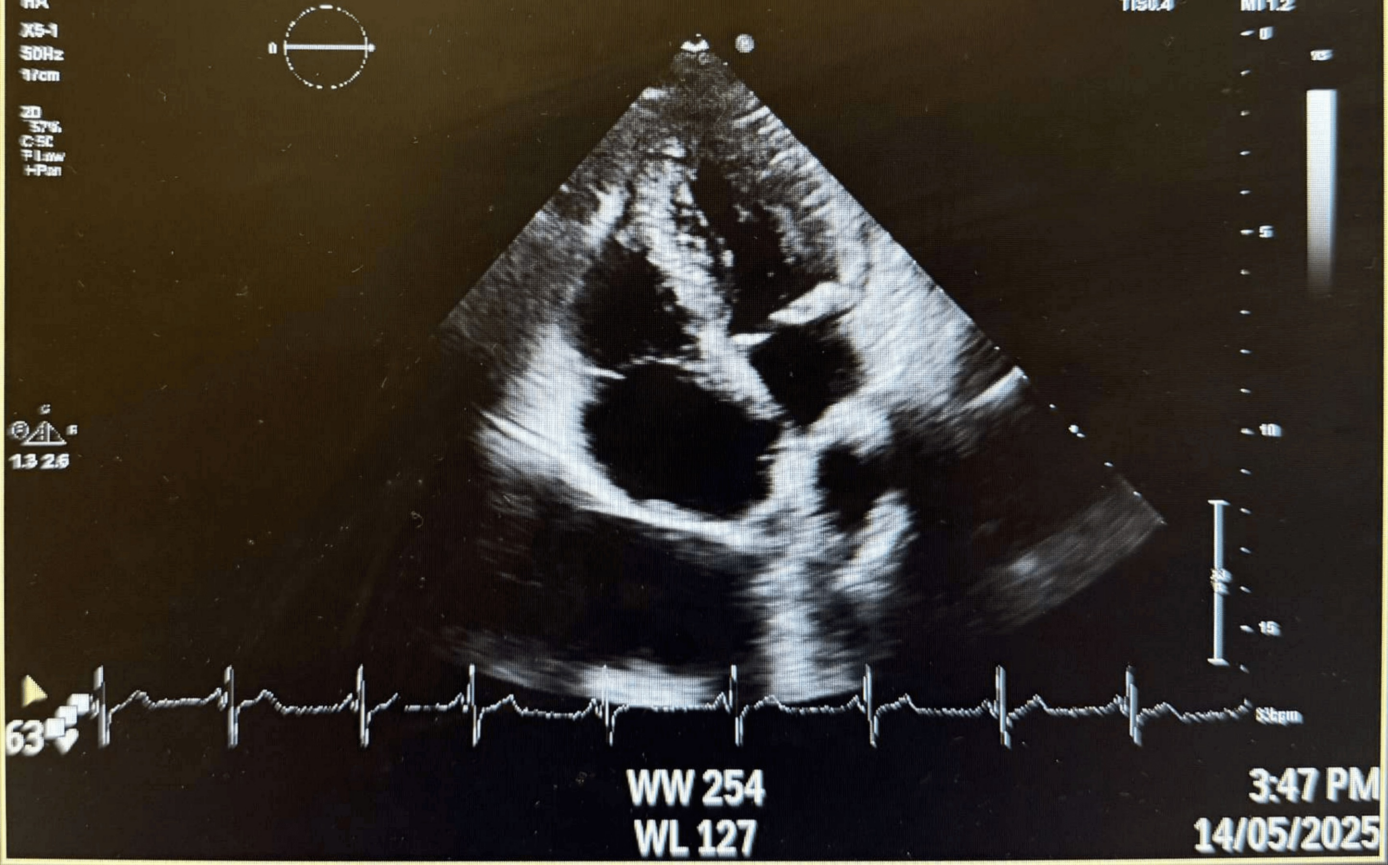
Bedside transthoracic echocardiography demonstrating preserved biventricular function with no intracardiac thrombus

Transthoracic echocardiography was performed to exclude a cardiac embolic source (Figure 1).

**Figure 2:**
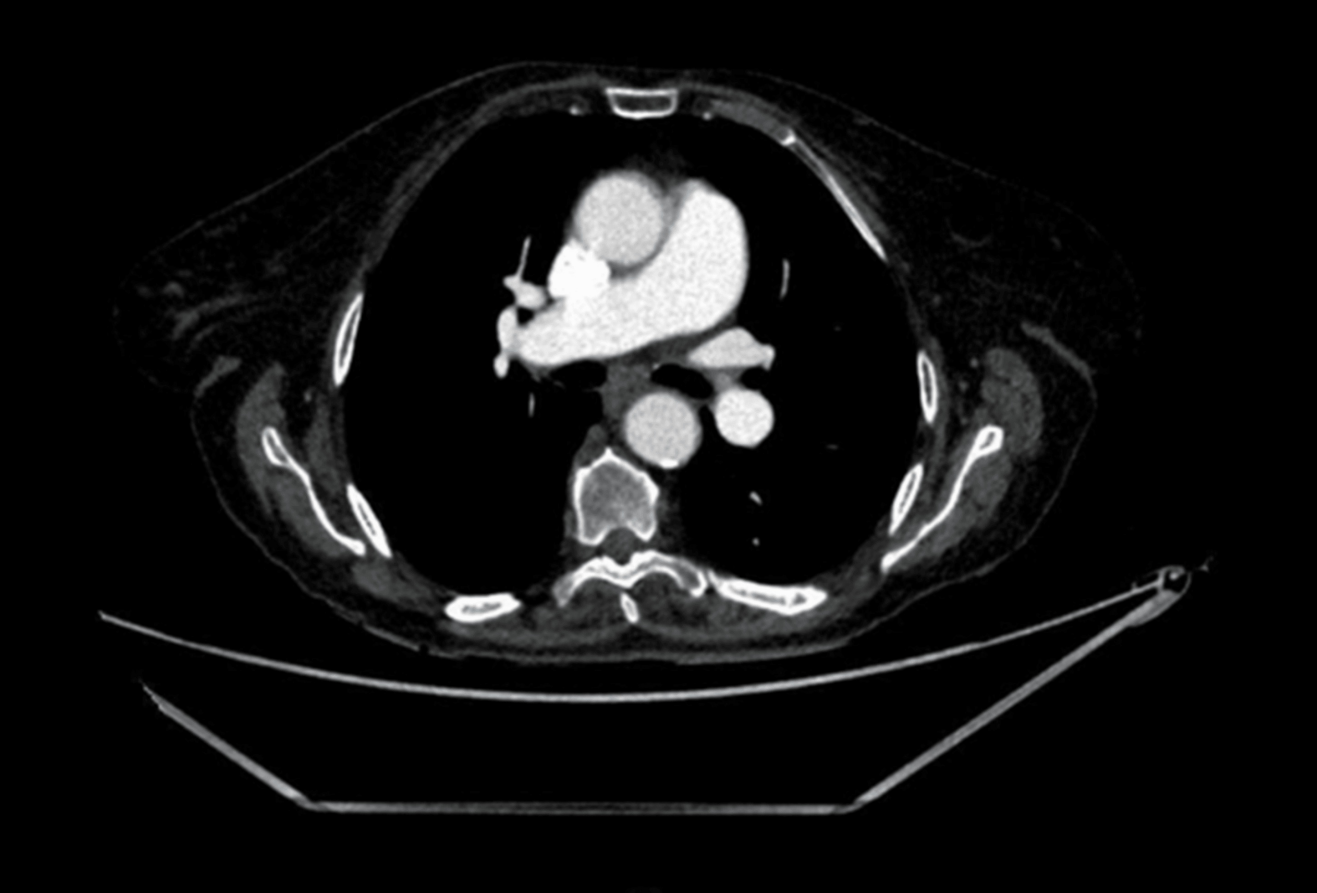
Contrast-enhanced CT angiogram demonstrating no evidence of large-vessel occlusion or alternative vascular cause of visual loss

CT angiography was performed and did not demonstrate any large-vessel occlusion or vascular cause of visual loss (Figure 2).

**Figure 3:**
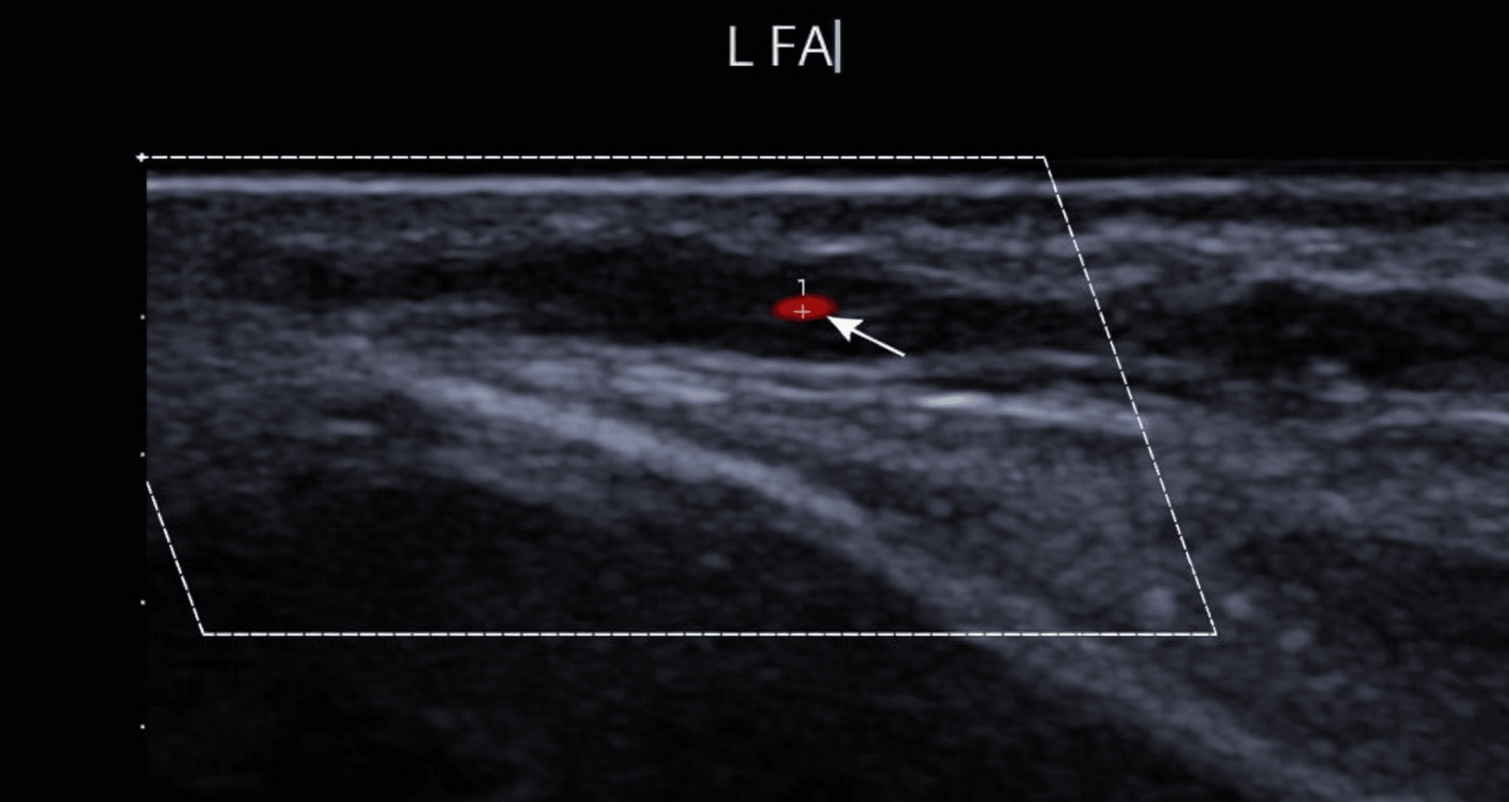
Temporal artery ultrasound demonstrating a halo sign consistent with giant cell arteritis

The case was discussed with the inpatient rheumatology team, who recommended urgent temporal artery ultrasound as the initial diagnostic investigation. Ultrasound demonstrated bilateral halo signs consistent with GCA (Figure 3). This supported the diagnosis and guided the path of treatment with corticosteroids.

**Figure 4:**
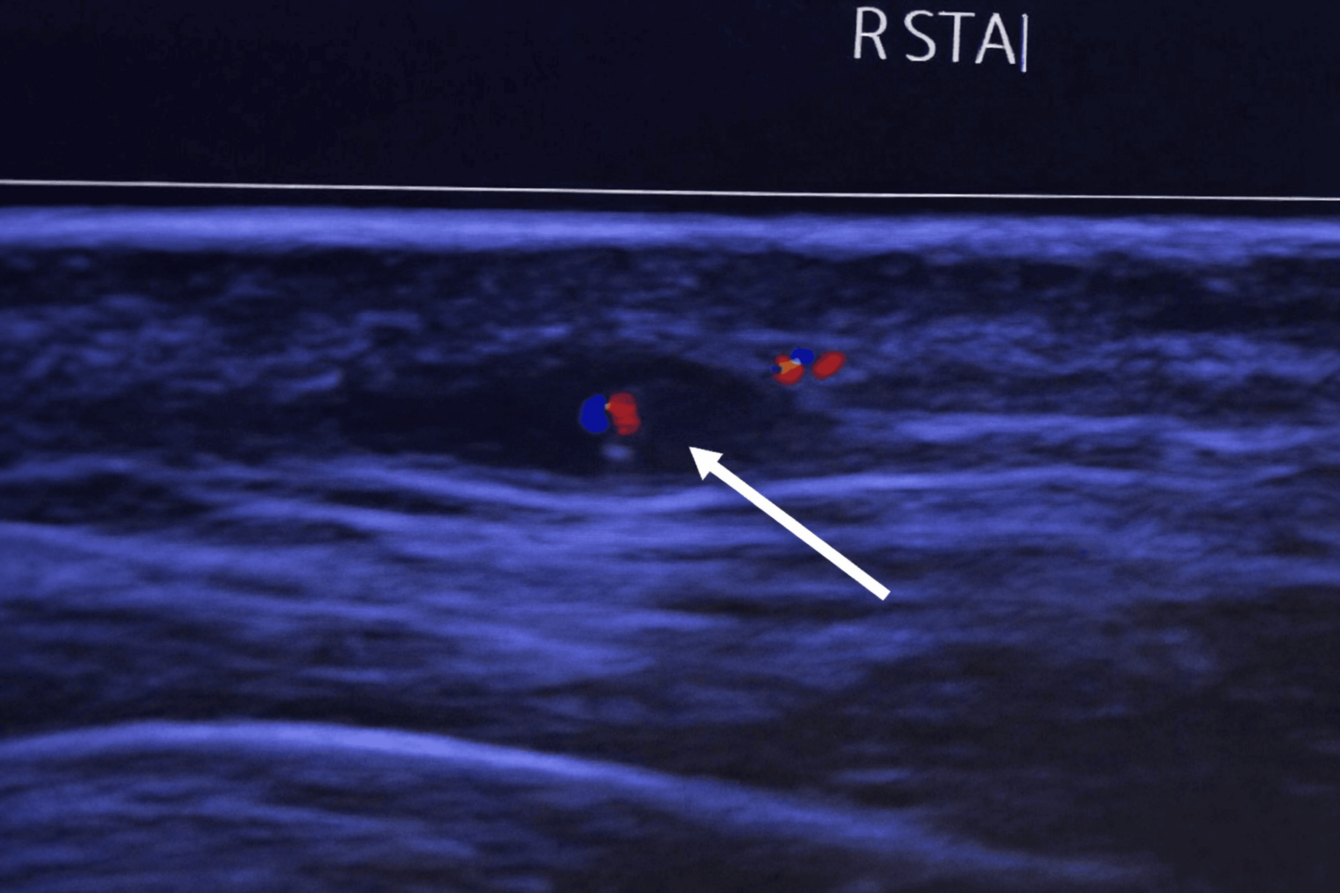
Ultrasound of the right superficial temporal artery demonstrating a circumferential hypoechoic halo sign (white arrow) consistent with active giant cell arteritis

Similar circumferential vessel wall thickening was observed in the right superficial temporal artery, confirming bilateral involvement (Figure 4).

Temporal artery biopsy was considered; however, following rheumatology review, ultrasound findings demonstrating bilateral halo signs were deemed sufficient to support the diagnosis, in keeping with current EULAR recommendations. Biopsy was therefore reserved for cases of diagnostic uncertainty.

Despite prompt initiation of corticosteroid therapy, the patient’s visual loss remained irreversible, consistent with established ischemic damage. During follow-up, the patient experienced recurrent inflammatory activity requiring escalation of corticosteroid therapy and multidisciplinary input. Repeat temporal artery ultrasound did not demonstrate active disease flare, supporting its role in disease monitoring.

The patient was discharged following multidisciplinary optimization, including physiotherapy and occupational therapy support. At outpatient follow-up, her symptoms remained stable, although inflammatory markers remained elevated, necessitating continuation of corticosteroid therapy. The case was discussed at a regional multidisciplinary meeting, and tocilizumab therapy was considered in the context of ongoing disease activity, pending exclusion of underlying infection. She is currently awaiting a CT of the chest, abdomen, and pelvis to exclude deep infection prior to initiation of tocilizumab therapy.

## Discussion

This case highlights an atypical presentation of GCA with rapidly progressive bilateral visual loss occurring in the absence of classic cranial symptoms, a recognized but diagnostically challenging scenario. Visual loss is a severe complication of GCA, most commonly due to anterior ischemic optic neuropathy or retinal artery occlusion, with delays in diagnosis substantially increasing the risk of irreversible blindness [2,3]. Elderly patients presenting without headache, scalp tenderness, or jaw claudication are therefore particularly vulnerable to delayed recognition.

Although temporal artery biopsy has traditionally been regarded as the diagnostic gold standard, it is invasive and limited by false-negative results due to skip lesions. In contrast, vascular ultrasound offers a reliable, non-invasive alternative, allowing real-time visualization of characteristic features, such as arterial wall edema and the hypoechoic halo sign, thereby facilitating earlier diagnosis and prompt initiation of treatment [4,5].

Evidence from meta-analyses and prospective studies has demonstrated that temporal artery ultrasound has good diagnostic accuracy and represents an effective alternative to biopsy in patients with suspected GCA. Previous reports have described both simultaneous and sequential involvement of the optic nerves, often resulting in poor visual outcomes. However, bilateral visual loss in the absence of preceding cranial or systemic symptoms remains uncommon. In this case, sequential visual loss without classical features contributed to diagnostic uncertainty and delay, ultimately resulting in irreversible visual impairment. This highlights the need for a high index of suspicion, particularly in atypical presentations.

The TABUL study demonstrated that ultrasound-based diagnostic pathways are both accurate and cost-effective, while the EUREKA study further validated the performance of vascular ultrasound in routine clinical practice across multiple centers [6,7]. In addition, quantitative ultrasound measures, such as the halo score, have been shown to correlate with disease severity and the risk of ocular ischemia, supporting their potential role in both diagnosis and disease assessment [8].

Consequently, current EULAR recommendations advocate ultrasound as the first-line imaging modality in patients with suspected GCA, reserving biopsy for cases where diagnostic uncertainty persists [1]. High-dose corticosteroids remain the cornerstone of treatment, with urgent initiation essential in sight-threatening disease. For patients with relapsing or refractory disease, interleukin-6 inhibition with tocilizumab has demonstrated efficacy in inducing sustained remission and reducing glucocorticoid exposure, as supported by randomized controlled trial data and NICE guidance [9,10].

## Conclusions

Clinicians should maintain a high index of suspicion for GCA in elderly patients with unexplained or progressive visual symptoms, even in the absence of classical cranial features. Temporal artery ultrasound is a non-invasive, cost-effective, and sensitive first-line investigation. While biopsy remains definitive, ultrasound’s practicality and multi-segment assessment make it invaluable. This case underscores that early recognition and prompt initiation of treatment are essential to prevent irreversible visual loss.
